# Supplementary material for: Causal interplay between lactose intolerance and gut microbiota: a combined bidirectional Mendelian randomization and in vivo validation study
Source: Front Nutr. 2026 Jun 1;13:1803337. doi: 10.3389/fnut.2026.1803337 (PMC13265576; doi:10.3389/fnut.2026.1803337)
Supplement: Supplementary file 1 [file Data_Sheet_1.zip › supplementary materials/Forward/leave one out cross validation/ebi-a-GCST90027579.finngen_R12_E4_LACTONAS.pdf]

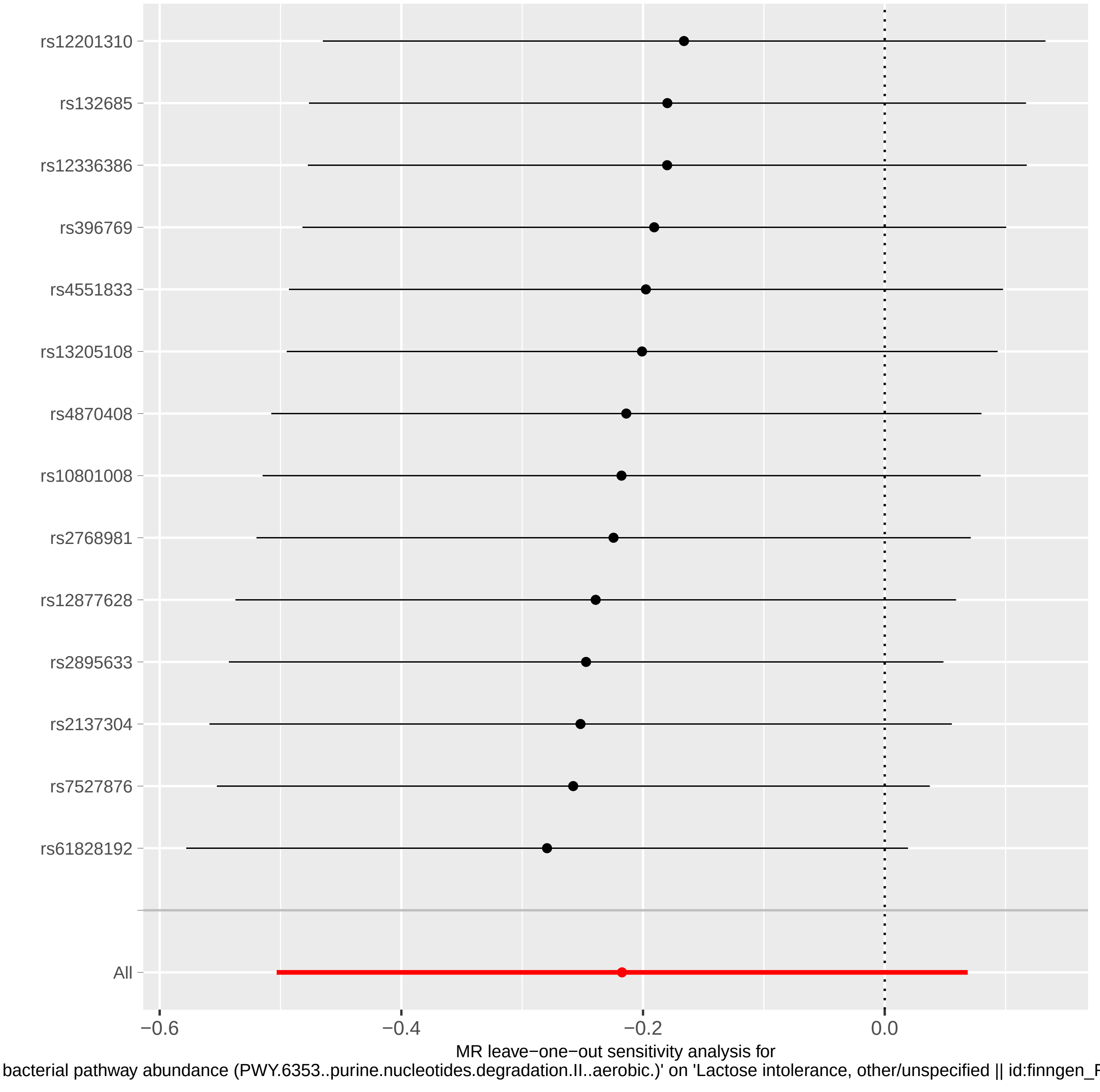

rs12201310
rs132685
rs12336386
rs396769
rs4551833
rs13205108
rs4870408
rs10801008
rs2768981
rs12877628
rs2895633
rs2137304
rs7527876
rs61828192
All
−0.6
−0.4
−0.2
0.0
MR leave−one−out sensitivity analysis for
bacterial pathway abundance (PWY.6353..purine.nucleotides.degradation.II..aerobic.)' on 'Lactose intolerance, other/unspecified || id:finngen_
